# Prevalence of cerebral small vessel disease in a Fabry disease cohort

**DOI:** 10.1016/j.ymgmr.2021.100815

**Published:** 2021-10-21

**Authors:** Daisy Tapia, David Floriolli, Eric Han, Grace Lee, Annlia Paganini-Hill, Stephani Wang, Setarah Zandihaghighi, Virginia Kimonis, Mark Fisher

**Affiliations:** a Division of Genetic and Genomic Medicine, Department of Pediatrics, University of California Irvine Medical Center, CA, USA; b Department of Radiological Sciences, Neuroradiology, University of California Irvine Medical Center, CA, USA; c Department of Neurology, University of California Irvine Medical Center, CA, USA; d Division of Cardiology, Department of Medicine, University of California Irvine Medical Center, CA, USA

**Keywords:** Fabry disease, Brain magnetic resonance imaging, Neurovascular disease, Cerebral small vessel disease

## Abstract

**Objective:**

To characterize the prevalence of brain ischemia and cerebral small vessel disease in a cohort of patients with Fabry disease (FD) seen at an academic medical center.

**Background:**

FD is an inherited X-linked lysosomal storage disorder with central nervous system involvement. Limited data are available in the literature on the cerebrovascular neuroimaging findings in FD, and the reported prevalence of stroke symptoms and cerebral small vessel disease has varied widely.

**Design/methods:**

Brain MRI was performed in 21 patients with FD followed at University of California Irvine Medical Center. Stroke symptoms were assessed and quantification of cerebral microvascular disease was performed using small vessel disease (SVD) score. Lacunes and deep white matter hyperintensities were scored on a four-point scale of 0 (absent) and 1–3 to account for increasing severity; microbleeds were scored 0 (absent) or 1 (present). The total SVD score is the sum of the three components and ranges from 0 to 7.

**Results:**

Nearly 43% (9/21) of our FD cohort (aged 32–81 years, mean = 50) had a SVD score of one or higher, all of whom were aged 50 or more years. The most common MRI-defined SVD was white matter hyperintensities (9/9, 100%), followed by microbleeds (6/9, 66%), and lacunes (3/9, 33%). The three patients with previous strokes had some of the highest SVD scores reported in the cohort (scores 3–5).

**Conclusions:**

In this cohort, the prevalence of SVD (43%) was three times higher than prevalence of stroke symptoms. SVD score was highest in the those who had experienced a stroke. These findings emphasize the importance of routine MRI screening of patients with FD in order to identify and treat high risk patients.

## 1 Introduction

Fabry disease (FD; OMIM 301500) is an X-linked disorder caused by mutations in the *GLA* gene which encodes for the enzyme alpha-galactosidase A, deficiency of which leads to accumulation of globotriaosylceramide (GL-3) within lysosomes. The reported incidence varies between 1 in 40,000 to 1 in 117,000, however the incidence may be much higher since the disorder is often missed, leading to delays in the diagnosis and treatment of the disease [[1], [2]]. The accumulation of GL-3 has widespread implications, as a variety of cell types are affected, including endothelial cells, podocytes, and cardiomyocytes [3]. Although the heart and kidneys are among the most commonly diseased organs in patients with this disease, cerebrovascular complications are also seen. Individuals with FD have a 12-fold increased risk of transient ischemic attack and stroke [4]. A review of cerebovascular involvement in FD reported stroke incidence varying from 24% to 48%, with the majority of strokes being ischemic [4]. Results from the Fabry outcome survey, based on registry data, reported the incidence of stroke in FD to be 13% with the mean age at first stroke earlier than that of the general population [5].

Cerebral small vessel disease (SVD) is well-characterized and magnetic resonance imaging (MRI) manifestations are a combination of small deep infarcts, cerebral microbleeds, white matter hyperintensities (WMH), and enlarged perivascular spaces [4,[6], [7]]. Cerebral SVD is common in aging adults, particularly after age 60 [[8], [9]]. However, the prevalence and severity of SVD in FD patients, particularly WMH, corresponds to individuals in the general population who are decades older [10].

An SVD scoring system quantifies cerebral SVD on a scale in which points are added for each detectable MRI feature associated with cerebral SVD. For each pathological MRI feature, a standardized definition, such as the Fazekas scale for WMH, or international consensus definition for microbleeds, is used [11]. The SVD scoring system provides a standardized and objective measurement of cerebral SVD severity [11]. This may be useful in exploring various relationships between FD and cerebrovascular pathology.

## 2 Materials and methods

### 2.1 Participants

Adult patients with FD followed clinically at University of California Irvine (UCI) Medical Center were recruited. Patients are seen once or twice a year as part of routine Fabry surveillance. UCI approved informed consent (HS 2008–6631) was obtained from all patients to collect and use data from clinical evaluations as part of the Fabry registry sponsored by Sanofi Genzyme. All patients had pathogenic variants in the GLA gene confirmed through molecular testing. Phenotype information (Supplemental Table 1) was sourced from the International Fabry Disease Genotype-Phenotype Database (dbFGP) [9].

### 2.2 Vascular risk factors and clinical features

Vascular risk factors and other clinical features were collected via review of electronic medical records. History of stroke was defined as clinically documented cerebral infarction with reported residual deficits. Diabetes mellitus was defined as Hgb A1c ≥ 6.5%, hypertension as blood pressure ≥ 140/90 mmHg, hyperlipidemia as combinations of two of: total cholesterol ≥200 mg/dL, low-density lipoprotein (LDL) ≥ 100 mg/dL, triglycerides ≥150 mg/dL, or high-density lipoprotein (HDL) ≤ 40 mg/dL. Coronary artery disease (CAD) and atrial fibrillation (AF) were defined as previously diagnosed by a physician and/or on current treatment. History of smoking was self-reported by patients and defined as having smoked at least 100 cigarettes in their entire lifetime.

### 2.3 Brain MRI

Brain MRIs were obtained as part of routine clinical care for FD management. Brain MRIs obtained at UCI (52%) were performed using susceptibility weighted imaging (SWI) with either 1.5 or 3 Tesla field strength. Assessments done at other institutions (48%) were performed using SWI or gradient recalled echo (GRE) and field strength, while generally 1.5 or 3 Tesla, was occasionally not indicated. Sequences included axial diffusion-weighted imaging (DWI), T2-weighted, fluid-attenuated inversion recovery (FLAIR) and sagittal T1-weighted sequences. Most patients had one MRI; for those with more than one we used their most recent assessment for the main analysis.

### 2.4 SVD scoring system

Brain MRIs were assessed for the presence of vascular lesions by an experienced neuroradiologist (DF) blinded to clinical information. Cerebral microvascular disease burden was quantified by calculating an SVD score [8] rating three independent variables: lacunes, deep WMH, and microbleeds. Lacunes and deep WMH were scored on four-point scale (0 for absent to 3) to account for increasing severity and microbleeds were scored 0 for absent 1 for present, leading to a total SVD score of 0–7.

### 2.5 Statistical analysis

Means and standard deviations of continuous variables and proportions of categorical variables are presented. Differences between groups were tested using Krushal-Wallis test for continuous variables and Fisher's exact test for categorical variables. Pearson and Spearman correlation coefficients were calculated for SVD score with age at MRI. Statistical analysis was performed using SPSS (IBM Corp. Released 2020. IBM SPSS Statistics for Windows, Version 25.0. Armonk, NY: IBM Corp).

## 3 Results

Twenty-one FD patients were included in this study. Table 1 summarizes the patients' demographics. The majority of participants were female (62%) and on Enzyme Replacement Therapy (ERT) (76%). Age at diagnosis ranged from 12 to 79 years (mean = 40) and age at MRI ranged from 32 to 81 years (mean = 50). Most participants (62%) had classic FD phenotype (Supplemental Table 1).

**Table 1:** Demographics of study sample of 21 participants with Fabry Disease.

Sex, *n* (%)	
Male	8 (38)
Female	13 (62)
Race, *n* (%)	
White	9 (43)
Hispanic	8 (38)
Asian	4 (19)
Age at MRI (years)	
Range	32–81
Mean ± standard deviation	50.0 ± 13.9
Age at diagnosis (years)	
Range	12–79
Mean ± standard deviation	40.2 ± 17.3
Age at initiation of enzyme replacement (years)	
*n* (%)	16 (76)
Range	17–65
Mean ± standard deviation	44.2 ± 15.2

### 3.1 Vascular risk factors

Table 2 gives the prevalence of vascular risk factors in the patients. Three participants (14%) had a previous stroke. Patient 17 experienced two strokes, the first at 53 years and the second at 60 years. The first was not well described but the second stroke resulted in cortical infarcts of the left parietal and occipital lobes. Patient 18 also experienced two strokes, the first at 56 years and the second at 62 years. The first was a small deep cerebellar infarction while the second showed left centrum semiovale multifocal ischemic injury. Patient 21 experienced a stroke at 80 years of age affecting the right occipital and parietal lobes. The most prevalent vascular risk factor was hyperlipidemia, present in 52%. Two patients (10%) had none of the vascular risk factors listed in Table 2. All but one patient was taking an antithrombotic medication, most often (90%) aspirin 81 mg, for stroke prevention. Individual patient risk factors are outlined in Supplemental Table 2. No pulvinar signs were observed in any of the 21 patients. Three patients (14%) had dolichoectasia, three were found in the vertebrobasilar system with one patient having addition dolichoectasia noted in the right IAC terminus. Two additional patients were found to have minimal basilar elongation, with neither ectasia nor dilatation.

**Table 2:** Vascular risk factors in study sample of 21 participants with Fabry Disease.

	Number (%)
Previous Stroke	3 (14)
Diabetes Mellitus	3 (14)
Hypertension	9 (43)
Hyperlipidemia	11 (52)
Coronary Artery Disease	2 (10)
Atrial Fibrillation	2 (10)
History of Smoking	3 (14)
Antithrombotic Medication*	20 (95)

Note: *Antithrombotic medication taken was 81 mg Aspirin (*n* = 18), 325 mg Aspirin (*n* = 1), Plavix (*n* = 1).

### 3.2 Total SVD score

**Fig. 1:**
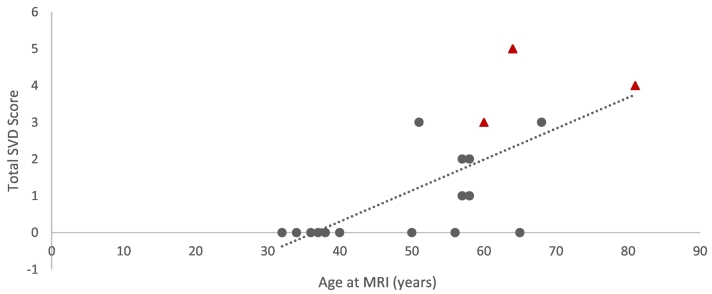
Plot of age at MRI against cerebral small vessel disease (SVD) score. Patients who had a previous stroke are indicated by red triangles.

**Fig. 2:**
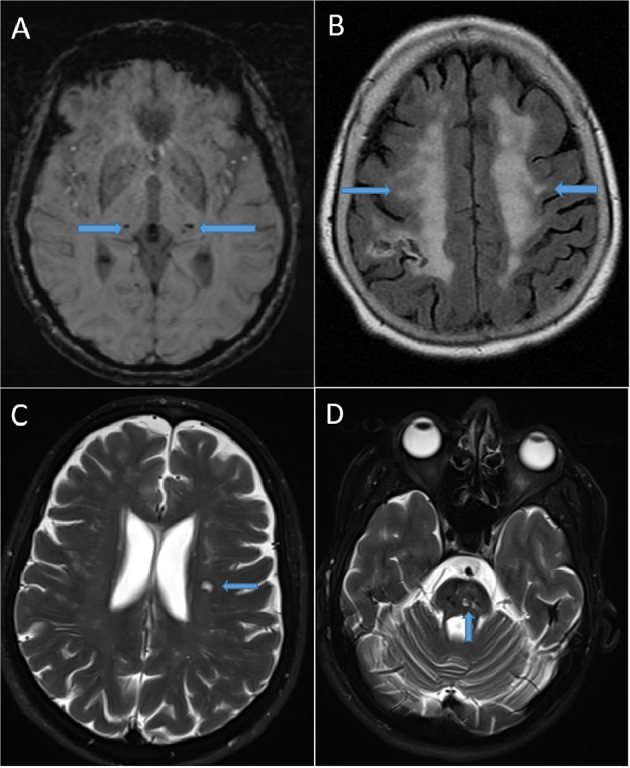
MRI examples of SVD features in Fabry Disease patient cohort. (A) Axial susceptibility weighted image shows the presence of bilateral thalamic microbleeds for an SVD microbleed score of 1 (microbleeds present) in 60-year-old male patient. (B) Axial FLAIR shows confluent subcortical white matter disease for a Fazekas score of 3 in 81-year-old female. A chronic right parietal infarction involving a portion of the right MCA territory is also partially imaged. (C) Axial T2 fat saturated image shows a pontine lacunar infarction in 64-year-old female. (D) Axial T2 fat saturated image in the same patient as image C shows another lacunar infarction in the left corona radiata. An additional lacunar infarction of the genu of the corpus callosum in this patient (not shown) results in an SVD lacunar score of 2 (3–5 lacunes).

**Fig. 3:**
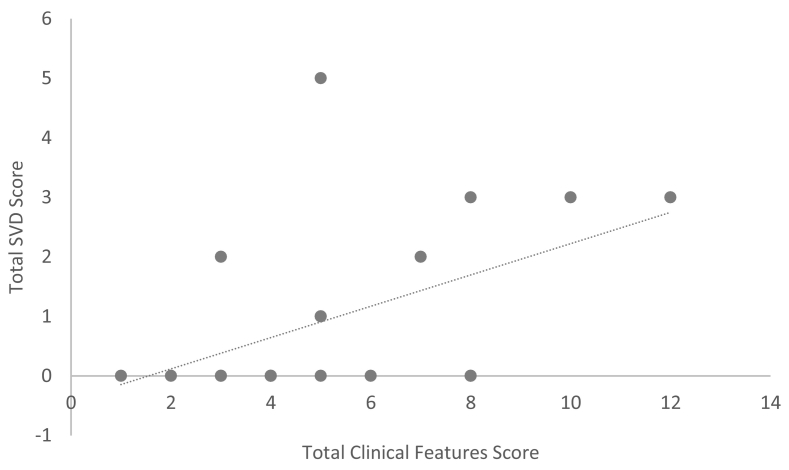
Expanded clinical features in study sample of 20 patients with Fabry Disease. Patient 21 was excluded from this analysis because she was lost to follow-up.

Nine participants (43%) had a SVD score of one or higher, all over the age 50 years (Table 3). The average SVD score was 1.1 (standard deviation = 1.6) for the entire sample and 2.0 (standard deviation = 1.7) for those aged 50 years and older. The total SVD score strongly correlated with age (Spearman correlation coefficient was 0.75, *p* value <0.001) as seen in Fig. 1. Mean SVD did not differ between the sexes or among the racial groups. The most common MRI-defined SVD was WMH (9/12), followed by microbleeds (6/12), and less frequently, lacunes (3/12). The three patients with previous stroke tended to have the highest SVD scores (scores 3, 4, and 5). Examples of SVD features on MRI are shown in Fig. 2. Only two patients had an SVD score of one, which was attributed to WMH. Individual patient SVD score details are outlined in Supplemental Table 3. The total SVD score also correlated significantly with the main clinical features associated with Fabry disease (Fig. 3, Supplemental Table 4).

**Table 3:** Distribution of cerebral small vessel disease (SVD) scores in study sample of 21 participants with Fabry Disease by age at diagnosis.

SVD score	< 50 years, *n* = 9 (%)	≥ 50 years, *n* = 12 (%)
0	9 (100)	3 (25)
1	0 (0)	2 (17)
2	0 (0)	2 (17)
3	0 (0)	3 (25)
4	0 (0)	1 (8)
5	0 (0)	1 (8)
6	0 (0)	0 (0)
7	0 (0)	0 (0)

### 3.3 Total SVD score removing patients with GRE MRI technology

Seven participants (33%) had brain MRIs performed on machines that used GRE sequences, which have lower sensitivity for detection of microbleeds. Excluding these, seven of the remaining 14 patients had an SVD score of one or higher; the average SVD score was 1.4 (standard deviation = 1.6); and the SVD score was strongly correlated with age (Spearman correlation coefficient was 0.64 with *p* value 0.01).

### 3.4 Patients with multiple MRI time points

Four patients in our cohort had an MRI scan previous to the one used in our analysis: 3, 4, 4, and 9 years earlier. The SVD scores were the same at both times except for the patient whose MRI results were 9 years apart. That patient's score was 4 at age 55 years and 5 at age 64 years, with a gain of one point in severity of lacunes. The patient's earlier MRI was performed using GRE sequencing whereas her most recent was done using SWI sequencing; the different sequences may have contributed to different findings.

## 4 Discussion

This study found that in FD patients the prevalence of cerebral SVD by MRI (43%) was three times higher than the prevalence of stroke symptoms (14%). These findings align with previous observations made in 51 patients with FD, 44% of whom had MRI evidence of SVD [13]. While previous studies have shown that SVD in the form of white matter lesions are predominant in the general population after the age of 60 years [9], we found that in our FD cohort the occurrence was evident one decade earlier at age 50. The observed association of SVD score with age has been reported in other cohorts as well [13,[15], [16]]. Additionally, 78% (7/9) of patients with a total SVD score greater than zero had findings other than WMH. These findings support the notion of a unique predisposition to SVD in patients with FD compared with the general population and support routine screening via brain MRI as part of FD care.

Our results showed that age was strongly associated with SVD score indicating an increased risk for developing stroke. It was notable that no one under the age of 50 years showed any cerebral SVD injury, particularly WMH, which are considered characteristic neuroradiological signs of FD. The published data regarding cerebrovascular findings in younger FD patients are limited. Available studies suggest that while cerebrovascular findings are less common in children and adolescents than in adults with FD, they are still prevalent [[12], [13]]. Sixteen percent of 44 children and adolescents with FD studied by Marchesoni et al. were found to have white matter lesions [12]. In another study, one child developed a hyperintense lesion in T2-weighted imaging in the left occipital region a year after a previously normal MRI and ERT initiation at age 12 years [13]. Nonetheless, our results are consistent with previous studies which have found WMHs present in almost half of patient with FD [ 10].

Our findings suggest that using an SVD score to characterize extent of SVD in FD patients might help better predict those at increased risk for developing a stroke. Current management guidelines recommend brain MRIs every 3 years as part of routine FD screening [3]. As it is challenging to note progression of cerebrovascular changes over time in an objective manner, providers may find benefit in using SVD scores to track alterations in MRI findings spanning various timepoints.

Although ERT has been shown to attenuate disease progression in the heart and kidney, its impact on cerebrovascular disease has not been demonstrated. Impact of ERT on stroke risk in our cohort is also unknown. In a previous study of 12 patients without prior stroke or ERT, three individuals had symptomatic strokes after ERT initiation, two within 6 months after initiation [14]. However, incidences of WMH or periventricular hyperintensities were not significantly different between the three individuals with stroke and those without [14].

Additionally, the use of antithrombotic medication, prescribed to minimize the risk of stroke in the general population, has not been studied in FD patients. Improved understanding of the impact of prophylactic agents on stroke risk for FD patients may lead to enhancement of preventative medicine via customization in medications for each patient.

Limitations of this study include a small sample size, although stroke prevalence in our cohort was similar to that reported in large registries. Additionally, not all MRIs were performed using the same scanner which led to a variation in field strength and sequences. Additional limitations include the paucity of serial imaging of our cohort. Patients should be encouraged to obtain brain MRI at the recommended 3-year intervals [3].

## 5 Conclusions

Although cerebral SVD tends to be ubiquitous in elderly adults, in our FD cohort we saw increased SVD scores in individuals aged 50–60-years, which is earlier than that expected solely based on age. These findings emphasize the importance of routine MRI screening of FD patients. Use of an SVD score may help identify patients who are more likely to develop stroke. Further research is needed in individuals with FD to determine more precisely the prevalence and severity of cerebral SVD and their association to clinical outcomes.

## Appendix A Supplementary data

Supplementary material Image 1

## References

[1] Desnick R.J. (2004). Enzyme replacement therapy for Fabry disease: lessons from two α-galactosidase A orphan products and one FDA approval. Expert. Opin. Biol. Ther..

[2] Poorthuis B.J., Wevers R.A., Kleijer W.J., Groener J.E., de Jong J.G., van Weely S. (1999). The frequency of lysosomal storage diseases in The Netherlands. Hum. Genet..

[3] Ortiz A., Germain D.P., Desnick R.J., Politei J., Mauer M., Burlina A., Eng C., Hopkin R.J., Laney D., Linhart A., Waldek S., Wallace E., Weidemann F., Wilcox W.R. (2018). Fabry disease revisited: management and treatment recommendations for adult patients. Mol. Genet. Metab..

[4] Kolodny E., Fellgiebel A., Hilz M.J., Sims K., Caruso P., Phan T.G., Politei J., Manara R., Burlina A. (2015 Jan). Cerebrovascular involvement in Fabry disease: current status of knowledge. Stroke.

[5] Mehta A., Ginsberg L., FOS Investigators (2005 Mar). Natural history of the cerebrovascular complications of Fabry disease. Acta Paediatr. Suppl..

[6] Giau V.V., Bagyinszky E., Youn Y.C., An S.S.A., Kim S.Y. (2019). Genetic factors of cerebral small vessel disease and their potential clinical outcome. Int J Mol Sci..

[7] Choi J.C. (2015). Genetics of cerebral small vessel disease. J. Stroke.

[8] Li Q., Yang Y., Reis C. (December 2018). Cerebral small vessel disease. Cell Transplant..

[9] de Leeuw F.E., de Groot J.C., Achten E., Oudkerk M., Ramos L.M., Heijboer R., Hofman A., Jolles J., van Gijn J., Breteler M.M. (2001 Jan). Prevalence of cerebral white matter lesions in elderly people: a population based magnetic resonance imaging study. The Rotterdam Scan Study. J. Neurol. Neurosurg. Psychiatry.

[10] Körver S., Vergouwe M., Hollak C.E.M., van Schaik I.N., Langeveld M. (2018 Nov). Development and clinical consequences of white matter lesions in Fabry disease: a systematic review. Mol. Genet. Metab..

[11] Staals J., Makin S.D., Doubal F.N., Dennis M.S., Wardlaw J.M. (2014). Stroke subtype, vascular risk factors, and total MRI brain small-vessel disease burden. Neurology.

[12] Marchesoni C., Cisneros E., Pfister P., Yáñez P., Rollan C., Romero C., Kisinovsky I., Rattagan L., León Cejas L., Pardal A., Sevlever G., Reisin R. (2018 Dec 15). Brain MRI findings in children and adolescents with Fabry disease. J. Neurol. Sci..

[13] Reisin R.C., Romero C., Marchesoni C., Nápoli G., Kisinovsky I., Cáceres G., Sevlever G. (2011 Jun 15). Brain MRI findings in patients with Fabry disease. J. Neurol. Sci..

[14] Yagita Y., Sakai N., Miwa K., Ohara N., Tanaka M., Sakaguchi M., Kitagawa K., Mochizuki H. (2019 Sep). Magnetic resonance imaging findings related to stroke risk in japanese patients with fabry disease. Stroke.

[15] Crutchfield K.E., Patronas N.J., Dambrosia J.M., Frei K.P., Banerjee T.K., Barton N.W., Schiffmann R. (1998 Jun). Quantitative analysis of cerebral vasculopathy in patients with Fabry disease. Neurology.

[16] Fellgiebel A., Müller M.J., Mazanek M., Baron K., Beck M., Stoeter P. (2005 Aug 23). White matter lesion severity in male and female patients with Fabry disease. Neurology.

