# Supplementary material for: Prevalence of cerebral small vessel disease in a Fabry disease cohort
Source: Mol Genet Metab Rep. 2021 Oct 21;29:100815. doi: 10.1016/j.ymgmr.2021.100815 (PMC8551215; doi:10.1016/j.ymgmr.2021.100815)
Supplement: Supplementary file 1 — Supplementary material [file mmc1.docx]

**Supplemental Table 1** *GLA* variant details in study sample of 21 patients with Fabry Disease

| Patient No. | Race | Nucleotide Change | Protein Change | Type | Phenotype |
| --- | --- | --- | --- | --- | --- |
| 16 | White | c.132G>T | W44C | Missense | Classic |
| 8,18 | **White** | **c.256T>C** | **Y86H** | **Missense** | **Classic** |
| 15 | Asian | c.427G>C | A143P | Missense | Classic |
| 2 | Hispanic | c.639+4A>T | IVS4+4A>T | Intronic | Classic |
| 13 | Hispanic | c.639+4A>T | IVS4+4A>T | Intronic | Classic |
| 12 | Asian | c.640-801G>A/ c.639+919G>A | IVS4+919G>A | Intronic | Later onset |
| 3,14 | Asian | c.640-801G>A/ c.639+919G>A | IVS4+919G>A | Intronic | Later onset |
| 4 | White | c.680G>A | R227Q | Missense | Classic |
| 5 | Hispanic | c.706T>C | W236R | Missense | Classic |
| 11 | White | c.816C>A | N272K | Missense | Classic |
| 6,9 | Hispanic | c.983G>T | G328V | Missense | Classic |
| 1 | White | c.1041_1042insG | A348GfsX27 | Small Insertion | Likely classic* |
| 20,21 | **Hispanic** | **c.1072_1074delGAG** | **E358del** | **Small Deletion** | **Classic** |
| 19 | Hispanic | c.1088G>A | R363H | Missense | Later onset |
| 17 | **White** | **c.1226_1231delCCACAG** | **P409_G411delinsR** | **Small Deletion** | **Classic** |
| 10 | White | c.1246C>T | Q416X | Nonsense | Classic |
| 7 | White | c.1250T>G | L417R | Missense | Classic |

Note: Phenotype data according to the International Fabry Disease Genotype-Phenotype Database (dbFGP) established and maintained by the Icahn School of Medicine at Mount Sinai. *New variant not found in database.

Patients who had a previous stroke are indicated by bold font.

**Supplemental Table 2** Vascular risk factor details in study sample of 21 patients with Fabry Disease

| Patient No. | Sex | Age at MRI | Previous Stroke | DM | HTN | HLD | CAD | AF | Hx Smoking | Risk Total |
| --- | --- | --- | --- | --- | --- | --- | --- | --- | --- | --- |
| 1 | M | 32 | - | - | - | - | - | - | - | 1 |
| 2 | F | 32 | - | - | + | - | - | - | - | 1 |
| 3 | F | 34 | - | - | - | - | - | - | - | 0 |
| 4 | F | 46 | - | - | - | - | - | - | - | 1 |
| 5 | M | 37 | - | - | + | + | - | - | - | 3 |
| 6 | F | 38 | - | - | - | - | - | - | - | 1 |
| 7 | F | 38 | - | - | - | + | - | - | - | 1 |
| 8 | F | 38 | - | - | - | + | - | - | - | 1 |
| 9 | M | 40 | - | - | + | + | - | - | + | 4 |
| 10 | F | 50 | - | - | - | + | - | - | - | 2 |
| 11 | M | 51 | - | - | + | - | - | - | + | 3 |
| 12 | M | 56 | - | - | - | - | - | - | + | 1 |
| 13 | F | 57 | - | + | + | + | - | - | - | 3 |
| 14 | F | 57 | - | - | - | - | - | - | - | 0 |
| 15 | F | 58 | - | - | - | - | - | - | - | 1 |
| 16 | F | 58 | - | - | - | + | - | - | - | 1 |
| 17 | **M** | **60** | **+** | **+** | **+** | **+** | **+** | **+** | **-** | **6** |
| 18 | **F** | **64** | **+** | **-** | **+** | **+** | **-** | **-** | **-** | **4** |
| 19 | M | 65 | - | + | + | + | - | - | - | 4 |
| 20 | M | 68 | - | - | - | - | - | + | - | 2 |
| 21 | **F** | **81** | **+** | **-** | **+** | **+** | **+** | **+** | **-** | **5** |

Abbreviations: AF = Atrial Fibrillation; CAD = Coronary Artery Disease; DM = Diabetes Mellitus; HLD = Hyperlipidemia; HTN = Hypertension; Hx = History.

Patients who had a previous stroke are indicated by bold font.

**Supplemental Table 3** Cerebral small vessel disease (SVD) score details in study sample of 21 patients with Fabry Disease

| Patient No. | Sex | Age at MRI | Field Strength | SWI/GRE | Microbleeds | Lacunes | WMH | Total SVD Score |
| --- | --- | --- | --- | --- | --- | --- | --- | --- |
| 1 | M | 32 | 1.5T | SWI | 0 | 0 | 0 | 0 |
| 2 | F | 32 | 1.5T | SWI | 0 | 0 | 0 | 0 |
| 3 | F | 34 | 1.5T | GRE | 0 | 0 | 0 | 0 |
| 4 | F | 46 | 3T | GRE | 0 | 0 | 0 | 0 |
| 5 | M | 37 | Unknown | GRE | 0 | 0 | 0 | 0 |
| 6 | F | 38 | 3T | SWI | 0 | 0 | 0 | 0 |
| 7 | F | 38 | 3T | SWI | 0 | 0 | 0 | 0 |
| 8 | F | 38 | Unknown | GRE | 0 | 0 | 0 | 0 |
| 9 | M | 40 | 1.5T | SWI | 0 | 0 | 0 | 0 |
| 10 | F | 50 | 3T | GRE | 0 | 0 | 0 | 0 |
| 11 | M | 51 | 3T | SWI | 1 | 1 | 1 | 3 |
| 12 | M | 56 | 1.5T | SWI+GRE | 0 | 0 | 0 | 0 |
| 13 | F | 57 | 3T | SWI | 1 | 0 | 1 | 2 |
| 14 | F | 57 | 1.5T | GRE | 0 | 0 | 1 | 1 |
| 15 | F | 58 | 3T | SWI | 1 | 0 | 1 | 2 |
| 16 | F | 58 | 1.0T | SWI | 0 | 0 | 1 | 1 |
| 17 | **M** | **60** | **1.5T** | **SWI** | **1** | **0** | **2** | **3** |
| 18 | **F** | **64** | **3T** | **SWI** | **1** | **2** | **2** | **5** |
| 19 | M | 65 | 3T | SWI | 0 | 0 | 0 | 0 |
| 20 | M | 68 | 3T | SWI | 1 | 0 | 2 | 3 |
| 21 | **F** | **81** | **1.5T** | **GRE** | **0** | **1** | **3** | **4** |

Note: Microbleeds are scored 0 for absent and 1 for present; lacunes and deep white matter hyperintensities (WMH) are scored on a four-point scale of 0 (absent) and 1 to 3 (accounting for increasing severity); leading to possible SVD score of 0 to 7. Patients who had a previous stroke are indicated by bold font.

**Supplemental Table 4** Expanded clinical features in study sample of 20 patients with Fabry Disease.

| Patient No. | Renal Involvement | Pulmonary Involvement | Cardiomyopathy | TIA/ Stroke | Angiokeratomas | Corneal Whorl | GI involvement | Lymphedema | Hypohidrosis | Acroparesthesia | Hearing Loss | Tinnitus | Total Clinical Features | Total SVD Score |
| --- | --- | --- | --- | --- | --- | --- | --- | --- | --- | --- | --- | --- | --- | --- |
| 1 |  |  |  |  | + | + |  |  |  | + |  | + | 4 | 0 |
| 2 |  |  |  |  | + |  | + |  |  | + |  | + | 4 | 0 |
| 3 |  |  |  |  | + |  |  |  |  |  |  |  | 1 | 0 |
| 4 |  |  |  |  |  | + | + |  | + | + |  |  | 4 | 0 |
| 5 |  |  | + |  | + | + | + | + |  | + | + | + | 8 | 0 |
| 6 |  |  |  |  |  | + | + |  |  | + |  | + | 4 | 0 |
| 7 |  |  |  |  | + | + |  |  |  | + |  |  | 3 | 0 |
| 8 | + | + |  |  |  | + | + |  |  | + |  | + | 6 | 0 |
| 9 | + |  |  |  | + | + | + | + |  | + | + | + | 8 | 0 |
| 10 |  |  |  |  |  |  | + |  | + | + |  |  | 3 | 0 |
| 11 | + |  | + |  | + | + | + | + | + | + |  |  | 8 | 3 |
| 12 |  |  |  |  |  |  | + |  | + |  |  |  | 2 | 0 |
| 13 | + | + |  |  | + | + | + |  |  | + | + |  | 7 | 2 |
| 14 |  |  |  |  | + | + |  |  | + | + | + |  | 5 | 1 |
| 15 |  |  |  |  | + | + |  |  | + |  |  |  | 3 | 2 |
| 16 |  |  |  |  | + | + | + |  |  | + |  | + | 5 | 1 |
| 17 | + | + | + | + | + | + | + | + | + | + | + | + | 12 | 3 |
| 18 |  |  |  | + | + |  |  |  |  | + | + | + | 5 | 5 |
| 19 | + |  |  |  |  |  |  |  | + | + | + | + | 5 | 0 |
| 20 | + |  | + |  | + | + | + | + | + | + | + | + | 10 | 3 |

Patient 21 was excluded from this analysis because she was lost to follow-up.
